# Giant condyloma acuminatum of the scrotum in a man with AIDS: a case report

**DOI:** 10.1186/1752-1947-5-272

**Published:** 2011-07-02

**Authors:** Peter M Nthumba, Peter Ngure, Patrick Nyoro

**Affiliations:** 1 Plastic, Reconstructive and Hand Surgery Unit, AIC Kijabe Hospital, Kijabe 00220, Kenya

## Abstract

**Introduction:**

Giant condyloma acuminatum, also called a Buschke-Löwenstein tumor, first described in 1925, is a slow-growing, locally aggressive, destructive tumor of the ano-genital region. Scrotal tumors are rare. Reports on giant condyloma acuminatum lesions in patients with HIV and AIDS are surprisingly even rarer.

**Case presentation:**

In this report, we present the case of a 42-year-old African man with AIDS who was undergoing anti-retroviral therapy. He was found to have a giant condyloma acuminatum of the scrotum. Wide surgical excision and scrotal reconstruction with a pedicled anterolateral thigh flap was performed, significantly improving his quality of life.

**Conclusion:**

Decision making regarding the goals of surgical intervention in the terminally ill is a complex process. The options include conservative medical palliation or palliative excision versus a curative excision that has the potential for significant morbidity. Wide surgical excision with local flap reconstruction significantly improved the quality of life of the patient described herein. The challenges presented by emerging or unusual presentations of surgical pathology secondary to HIV and AIDS in patients who are on anti-retroviral therapy provide an opportunity for research and the establishment of guidelines for the use of adjuvant chemotherapy in these patients.

## Introduction

Giant condyloma acuminatum, also known as Buschke-Löwenstein tumor, was first described by Buschke and Löwenstein in 1925. This slow-growing, locally destructive tumor of the ano-genital region is thought to be induced by human papillomavirus (HPV), most commonly HPV types 6 and 11 and occasionally types 16 and 18. It is associated with extensive local infiltration and a high propensity to recur. Most authors consider it to be a verrucous carcinoma, a variant of squamous cell carcinoma that seldom metastasizes [1,2]. Histological examination, however, may reveal pockets of squamous cell carcinoma, a risk factor for metastasis. Giant condyloma acuminatum most often affects the glans penis, but has also been reported in the scrotum, vulva, the peri-anal region, ano-rectum and the bladder [3].

Scrotal giant condyloma acuminatum tumors are rare. While ano-genital condylomata acuminatum or warts are common lesions in patients with HIV, giant condyloma acuminatum and Buschke-Löwenstein tumors in patients with HIV are very rare [4,5].

In this report, we describe the case of a patient with AIDS undergoing anti-retroviral (ARV) therapy who presented to our hospital with a giant condyloma acuminatum lesion of the scrotum, and we explore the therapeutic difficulties posed by his care.

## Case presentation

**Figure 1:**
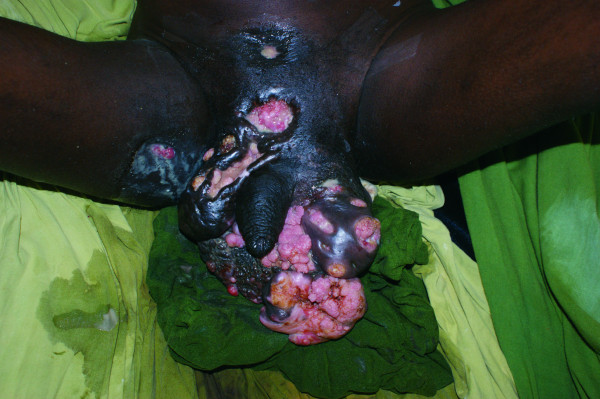
**Giant condyloma acuminatum of the scrotum**. Note multiple ulcers and discharging sinuses on the scrotum, thigh, and suprapubic areas.

A 42-year-old African man was referred to our institution from another country with a large scrotal tumor first noted five years prior to his presentation to our institution. The mass had grown slowly over the years, with ulceration associated with foul-smelling discharge noted with time, along with seepage of urine from multiple fistuli in the growing scrotal mass (Figure 1). He had been diagnosed with HIV, and ARV therapy had been started two years prior to the development of the scrotal mass. He also had the wasting syndrome, an AIDS-defining diagnosis. He had been unable to walk for three years prior to his visit to our institution. He had been married, but was separated from his wife upon developing the scrotal tumor.

The patient's physical examination revealed that he was emaciated. He had a large scrotal mass that extended laterally into both thighs, superiorly into the supra-pubic region and posteriorly to the anal verge. His hips had a fixed flexion and abduction deformity.

**Figure 2:**
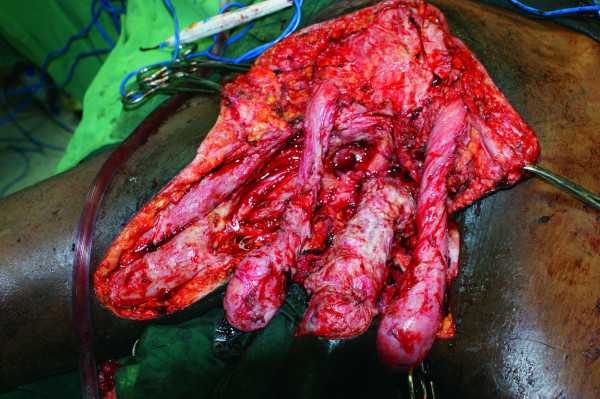
**Post-resection extent into both thighs and deep margins defined by the rectus sheath superiorly and the pubic arches and symphysis**. The proximal penile shaft and both testicles are completely exposed.

His hemoglobin was 7.4 g/dL, and his CD4 lymphocyte count was 150 cells/mm^3^. During surgery, the tumor was found to have extended laterally into the adductor musculature bi-laterally, requiring their inclusion in the excision. The deep margins included the rectus muscle fascia, pubis, pelvic diaphragm, and rectum (Figure 2). A small part of the bulbous urethra was excised with the tumor. The excised tumor weighed 1800 g and was reported on the basis of a histological examination to be a giant condyloma acuminatum.

**Figure 3:**
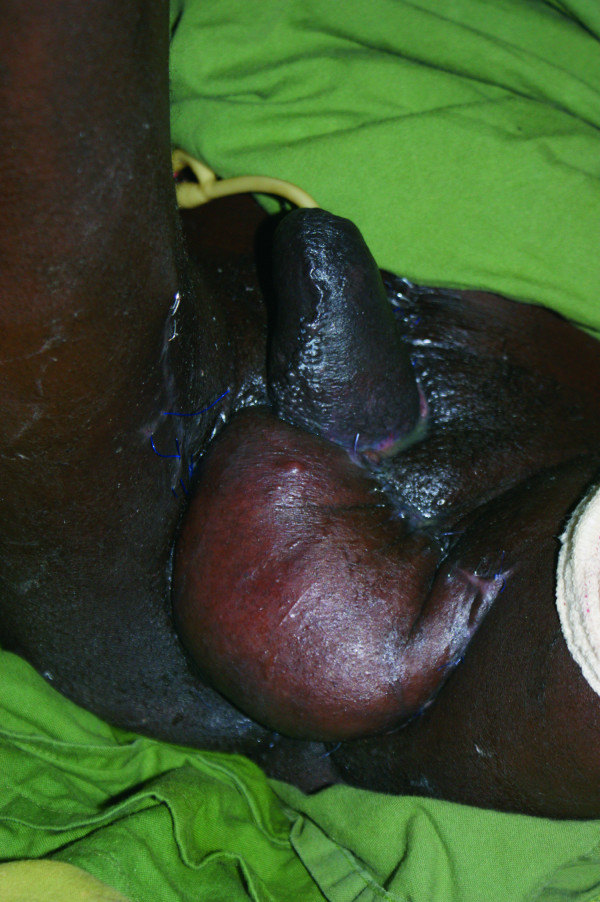
**Six weeks post-resection pedicled anterolateral thigh flap reconstruction of scrotum and perineum is shown**.

A pedicled left anterolateral thigh flap was used to create a neo-scrotum as well as to cover the resulting thigh defects (Figure 3). Negative pressure therapy was used to exclude the surgical wounds from fecal matter as well as to assist in draining the extensive surgical field. The patient recovered and was discharged to home with a urinary (urethral) catheter.

One month after discharge from the hospital, his wounds were healed, except for a tiny wound in the suture line of his neo-scrotum. His catheter accidentally fell off, and he was noted to be leaking urine through the wound previously noted. A supra-pubic cystostomy was performed and a catheter was placed, and the perineal urine leak was noted to have stopped a few weeks later. Six months post-operatively, some tumor recurrence was noted and discussions of chemotherapy were held but were not implemented because of the potentially complex interaction between the chemotherapy, ARVs, and the patient's precarious physical status.

## Discussion

Giant condyloma acuminatum generally occurs in adults, but has also been reported in children [6]. Poor penile hygiene is a known predisposing factor, while chronic inflammation (peri-anal fistuli), immunosuppression (HIV or therapy), diabetes, pregnancy, poor socioeconomic status, and smoking are recognized risk factors.

The patient presented herein had not been circumcised (Figure 1) and was also immunosuppressed, with a low CD4 count, despite having been on ARVs for a long period. Although immunosuppression is a recognized risk factor, giant condyloma acuminatum in the background of HIV/AIDS and ARV therapy is extremely rare and poses difficulties in decision making. How radical should surgical extirpation be? How aggressive should any adjuvant therapy be? The use of ARVs and immunosuppressants and/or immunomodulators is established in the care of renal transplant patients with HIV infection. A meta-analysis by Landin et al. of 12 series of reports of HIV-positive recipients under HAART receiving kidney transplants [7] reported the development of an AIDS-defining tumor in a single patient. A conclusion of this meta-analysis was that renal transplantation, and therefore the use of immunosuppressive therapy, is safe in patients with HIV undergoing highly active anti-retroviral therapy (HAART). There have been no reports on the use of immunosuppressive therapy in patients with AIDS who are being treated with ARVs, however.

The rarity of giant condyloma acuminatum in patients with HIV/AIDS is puzzling, considering that HPV is known to be the underlying cause of this lesion and also underlies cervical cancer, an AIDS-defining cancer, and anal cancer, a non-AIDS-defining cancer. The incidence of these lesions has not been affected by the use of ARVs. A possible explanation may be that HAART has largely eliminated giant condyloma acuminatum, a clinically benign tumor. This is conceivable, given the biological differences between giant condyloma acuminatum and squamous cell carcinoma. Under-reporting of cases of giant condyloma acuminatum with underlying HIV/AIDS is the other possible explanation for the paucity of literature on this rare condition.

Wide surgical excision, radiochemotherapy, topical and intra-lesional chemotherapy, carbon dioxide laser therapy, and photodynamic therapy have all been used in different combinations in the treatment of giant condyloma acuminatum, with varying success. Tytherleigh *et al. *[8] reported the successful use of neo-adjuvant chemoradiotherapy to down-size a tumor with subsequent complete surgical excision. The administration of an autogenous vaccine after surgical excision has the lowest reported recurrence rates at one year (less than 5%) [9]. There is a risk of transformation of a giant condyloma acuminatum into an aggressive squamous cell carcinoma (30% to 56% over five years), in addition to a 10% risk of anaplastic transformation after radiotherapy [1,6].

Surgery remains the most effective mode of management. In our patient, wide excision of the tumor was performed, but the deep perineal margins were difficult to obtain as this would have required a total penectomy, bilateral pubectomy, and resection of the pelvic diaphragm, an extensive procedure with significant morbidity that was considered unwarranted in a patient in whom the primary goal was palliation, considering his diagnosis of AIDS. Total penectomy and perineal urethrostomy have been reported previously [3].

Post-operatively, our patient was able to ambulate and perform a number of activities of daily living; the foul-smelling exudate and pain were gone. Upon discharge, his ability to feed, wash, clean himself after a bowel movement, and dress himself were very rewarding.

Surgical excision is the primary mode of treatment of giant condyloma acuminatum. Recurrence rates of 50% have been reported [1]. Tumor recurrence in our patient was noted six months post-operatively. The patient's improved nutritional status and motivation may warrant a revision of the original intent of the surgery, subject to the patient's consent, allowing an aggressive surgical excision. There is as yet no evidence of the efficacy of immunosuppressive therapy administered to patients with AIDS receiving ARV therapy. Certainly the challenges presented by emerging or unusual presentations of surgical pathology secondary to HIV/AIDS offer a challenge and indicate the need to establish guidelines on the use of chemotherapeutic agents, in addition to ARVs, in this patient population [10,11].

The patient reported herein recovered well after extensive surgery, despite his compromised immune status in an environment with limited resources. The case represents a challenge in clinical decision making when faced by scenarios such as we did in this case: conservative palliation (which would have condemned our patient to continued suffering) versus surgical palliation (which offered the opportunity for an improved quality of life but placed the patient and caregivers at potential risk).

## Conclusion

Giant condyloma acuminatum, rarely reported in patients with HIV/AIDS, is a rare, locally aggressive tumor that may recur early after surgical excision. Decision making regarding the goals of surgical intervention is complex and involves palliative excision versus a curative excision that has the potential for significant morbidity in a patient who is terminally ill. Wide surgical excision with local flap reconstruction significantly improved the quality of life of the patient presented here.

The challenges of treating patients with emerging or unusual presentations of surgical pathology secondary to HIV/AIDS who are receiving ARV therapy provide the opportunity for research and the establishment of guidelines on the use of adjuvant chemotherapy.

## Abbreviations

AIDS: acquired immunodeficiency syndrome; ARV: anti-retroviral; HAART: highly active anti-retroviral therapy; HIV: human immunodeficiency virus; HPV: human papillomavirus.

## Consent

Written informed consent was obtained from the patient for publication of this case report and any accompanying images. A copy of the written consent is available for review by the Editor-in-Chief of this journal.

## Competing interests

The authors declare that they have no competing interests.

## Authors' contributions

PMN, PNg, and PNy came up with the idea of writing the manuscript. PMN, PNg, and PNy made significant contributions to the writing of the manuscript. PMN provided leadership in writing the manuscript. All authors read and approved the final manuscript.
